# Resistance of *Borrelia burgdorferi* Sensu Lato Isolates from Serbia to Human Complement

**DOI:** 10.3390/pathogens15070723

**Published:** 2026-07-09

**Authors:** Gorana Veinović, Sanja Ćakić, Darko Mihaljica, Ratko Sukara, Aleksandra Stefanović, Elizabeta Ristanović, Eva Ružić-Sabljić, Snežana Tomanović

**Affiliations:** 1 Group for Medical Entomology, Centre of Excellence for Food and Vector-Borne Zoonoses, Institute for Medical Research, National Institute of Republic of Serbia, University of Belgrade, 11102 Belgrade, Serbia; darko.mihaljica@imi.bg.ac.rs (D.M.); ratko.sukara@imi.bg.ac.rs (R.S.); snezanat@imi.bg.ac.rs (S.T.); 2 Faculty of Biology, University of Belgrade, 11158 Belgrade, Serbia; sanjacakic83@yahoo.com; 3 Department of Medical Biochemistry, Faculty of Pharmacy, University of Belgrade, 11221 Belgrade, Serbia; aleksandra.stefanovic@pharmacy.bg.ac.rs; 4 Institute for Microbiology, Military Medical Academy, University of Defense, 11040 Belgrade, Serbia; elizabeta.ristanovic@vma.mod.gov.rs; 5 Laboratory for Diagnostics of Borreliosis and Leptospirosis, Institute for Microbiology and Immunology, Faculty of Medicine, University of Ljubljana, 1000 Ljubljana, Slovenia; eva.ruzic-sabljic@mf.uni-lj.si

**Keywords:** *Borrelia*, human complement, resistance

## Abstract

Serbia is characterised by the high prevalence and diversity of *Borrelia burgdorferi* sensu lato strains in ticks, but the reported incidence of Lyme borreliosis is much lower than that in regions with a similar prevalence of *Borrelia* in ticks. To evaluate the susceptibility and viability profiles of *Borrelia* strains circulating in local enzootic cycles, we analysed their response to human complement in vitro using short-term incubation periods. A total of 31 strains, comprising 27 Serbian tick isolates, two tick isolates from Spain, one tick isolate from Portugal, and one human skin isolate from Portugal (12 *Borrelia afzelii*, 12 *Borrelia lusitaniae*, three *Borrelia bavariensis*, two *Borrelia garinii*, and two *Borrelia valaisiana*), were analysed using serum susceptibility testing. The motility of spirochetes was assessed one and three hours after incubation with normal human serum (NHS) and heat-inactivated serum (HIS) used as a control. The absence of a statistically significant decrease in strain motility after incubation with NHS compared with HIS indicated the resistance of the strains. All tested *B. afzelii* and *B. bavariensis* strains and one *B. valaisiana* strain were serum-resistant, whereas both *B. garinii* strains were serum-susceptible. Based on statistical analysis, all *B. lusitaniae* strains were classified as serum-susceptible; however, two local tick isolates and the human reference strain demonstrated distinct biological heterogeneity, maintaining significantly higher residual motility after short-term exposure to human serum, which underscores strain-specific and geographic variations in complement tolerance.

## 1. Introduction

Lyme borreliosis (LB), a tick-borne infectious disease caused by spirochetes of the *Borrelia burgdorferi* sensu lato (s.l.) complex, is the most widespread vector-borne disease in the Northern Hemisphere [1,2]. Currently, 21 described *Borrelia* species with considerable variability in hostvector associations and geographical distribution are assigned to the *B. burgdorferi* s.l. complex [3]. Many different molecular methods are available for *Borrelia* species genotyping. They are either PCR-based typing methods (sequencing, restriction, Tm determination of PCR product, etc.) or whole-genome-based typing methods (pulsed-field gel electrophoresis, plasmid profile analysis, whole-genome sequencing) [4]. Different molecular typing methods vary in their taxonomic resolution and discriminatory power. In general, methods based on whole-genome typing and multilocus sequence typing have the highest resolution, and they can be used for the differentiation of *B. burgdorferi* s.l. among and within species [5]. Only a subset of *B. burgdorferi* s.l. species are known to cause diseases in humans [6]. In North America, LB is predominantly caused by *Borrelia burgdorferi* sensu stricto (s.s.), whereas in Europe, the overall diversity of *B. burgdorferi* s.l. is higher, and LB is caused by *Borrelia afzelii*, *Borrelia garinii*, *Borrelia bavariensis* (formerly *B. garinii* OspA serotype 4), *Borrelia spielmanii*, and, more rarely, *Borrelia burgdorferi* s.s.; *Borrelia valaisiana*, *Borrelia lusitaniae*, and *Borrelia bissettii* have only been identified in isolated cases, with their pathogenic relevance remaining a subject of ongoing debate [1,2]. Infection with different *Borrelia* species leads to different clinical manifestations, where the skin lesion erythema migrans is the most common one [1,2]. In persistent infection, *B. afzelii* is mostly associated with skin manifestations; *B. garinii* and *B. bavariensis* are usually associated with nervous system disorders; and *B. burgdorferi* s.s. is often associated with the development of arthritis [1,2,7,8].

Complement is a central part of innate immunity that serves as a first line of defence against foreign cells and pathogens [9]. As a survival strategy that leads to the establishment of an infection in the host, microorganisms have developed mechanisms to evade complement attack [9,10]. The characteristic serum resistance pattern raises the possibility that complement contributes to *Borrelia* transmissibility, host adaptation, and dispersal of spirochetes in nature; thus, it plays an important role in *Borrelia* ecology [10].

In vitro studies have shown that *B. afzelii*, *B. bavariensis*, *B. spielmanii*, *B. burgdorferi* s.s., and *Borrelia japonica* are resistant to human complement, while *B. garinii*, *B. valaisiana*, and *B. lusitaniae* are highly susceptible to human complement [11,12]. Differences in serum susceptibility have also been found among strains of the *B. valaisiana* species and *B. garinii* species [13,14].

While *B. valaisiana*, *B. afzelii*, and *B. garinii* are the most prevalent species in ticks throughout Europe [15], studies on the presence of *B. burgdorferi* s.l. in ticks from Serbia [16,17] have indicated the dominance of *B. lusitaniae*, followed by *B. afzelii*, *B. bavariensis*, *B. garinii*, *B. valaisiana*, and *B. burgdorferi* s.s. The prevalence of *B. burgdorferi* s.l. in *Ixodes ricinus* ticks in Serbia varies from 21.1% to 42.5%, depending on the region and detection methods [17]. While the region of Serbia and the Balkan Peninsula is characterised by the high prevalence and diversity of *Borrelia* species in *I. ricinus* ticks [18], the reported annual incidence of LB in Serbia between 2013 and 2019 remained comparatively low, ranging from 6.83 to 13.32 per 100,000 inhabitants [19].

This stands in sharp contrast to other European regions with similar tick infection dynamics [20,21]. For instance, in Germany, Slovenia, and Austria, annual incidence rates are among the highest in Europe, reaching 111/100,000 [20], 188.7/100,000 [20], and approximately 135/100,000 [22], respectively. This hyperendemic pattern is also observed in Estonia and Switzerland, where national incidence rates frequently surpass 100 cases per 100,000 inhabitants [22]. Furthermore, high rates are documented in Lithuania (99.9/100,000) [21] and Finland (61/100,000) [21], while countries like the Czech Republic consistently report heavy disease burdens, ranging between 27.6 and 46.1 cases per 100,000 inhabitants [23]. In comparison, the annual incidence rate in France between 2009 and 2017 was 53/100,000 [21], and primary care data from the United Kingdom estimated an annual incidence rate reaching 12.1/100,000 [24], all of which remain notably elevated above the baseline numbers recorded in Serbia [19].

The most common clinical manifestation of LB in Serbia is erythema migrans, which occurs in 93.21% of cases, followed by neurological, musculoskeletal, and cardiac manifestations in 2.80, 2.46, and 1.10% of cases, respectively [25], but there are no data on which *Borrelia* species cause certain clinical manifestations of LB, and isolates from human materials are lacking at present. However, apart from several studies on the presence and diversity of *B. burgdorferi* s.l. in ticks and animal hosts [17], there are no data on the exact *Borrelia* species that cause LB in humans.

Considering the epidemiological context in this region, the objective of this study was to evaluate the in vitro susceptibility and viability profiles of local vector-derived *Borrelia* strains to human serum. Furthermore, as *B. lusitaniae* represents the predominant species in both Serbia and Mediterranean countries, we aimed to compare the time-dependent survival profiles of available *B. lusitaniae* strains from Serbia, Spain, and Portugal in human serum to investigate potential geographic variations in their susceptibility in vitro.

## 2. Materials and Methods

### 2.1. Borrelia Strains

A total of 27 *Borrelia* strains (12 *B. afzelii*, 8 *B. lusitaniae*, 3 *B. bavariensis*, 2 *B. garinii*, and 2 *B. valaisiana* strains) were isolated from *I. ricinus* ticks from different localities in Serbia; these were previously described by Ćakić and colleagues [16]. Four external *B. lusitaniae* strains (Heavy, Listu, PotiB2, and PoHL1) were isolated from *I. ricinus* ticks and human skin. The isolation of *B. lusitaniae* (PoHL1) from the skin sample used in this study was previously described by Collares-Pereira and colleagues [26]. All *Borrelia* strains from Serbia were isolated in 2013 and 2014 [16], while *B. lusitaniae* strains from Spain and Portugal were isolated earlier (Listu, Heavy, and PoHL1 strains were isolated from 2000 to 2006, while the PotiB2 strain was isolated in 1993) [26,27,28,29]. Isolates from Spain and Portugal were later thoroughly described and comprehensively characterised [30]. Although the multi-decadal chronological gap between strain isolations could introduce a risk of degradation or plasmid loss during long-term storage, this factor was carefully mitigated. All cultures were stored appropriately, according to the recommendations for long-term storage and preservation of *Borrelia* cultures (at −80 °C (CryoCube F570, Eppendorf, Hamburg, Germany) in a medium containing Brucella broth (Becton, Dickinson and Company, Sparks, MD, USA) and 15% glycerol (Sigma-Aldrich Chemie GmbH, Steinheim, Germany)), and all tested strains were of low-passage status. Specifically, the low-passage status ranged from 2 to 5 passages for Serbian isolates and did not exceed 7 passages for the isolates from Spain and Portugal, with the exact passage number for each strain provided in Table 1. Furthermore, strains were revived from separate, single-use frozen vials to avoid repeated freeze–thaw cycles. Consequently, any storage-induced divergence was successfully minimised, ensuring robust comparability between the geographical groups. Prior to performing the serum susceptibility tests, the strains were re-cultivated from the biobank stocks under aseptic conditions provided by a laminar flow box. Briefly, the cryopreserved spirochetes were thawed at room temperature, and 1 mL of each culture (with an initial density of ~1 × 10^7^ cells/mL) was inoculated into sterile glass tubes (Sigma Aldrich, Steinheim, Germany) containing 6 mL of commercial, complete Barbour–Stoenner–Kelly–H (BSK-H) medium (Sigma-Aldrich, St. Louis, MO, USA) without the addition of antibiotics. To achieve precise experimental coordination and manage the workload effectively, the 31 strains were revived and tested in successive batches rather than simultaneously. To maintain the necessary microaerophilic conditions for optimal spirochete growth, the glass tubes were tightly sealed with rubber stoppers. The cultures were then incubated at 33 °C (Heratherm IGS180, Thermo Scientific, Waltham, MA, USA) and monitored via dark-field microscopy (BX41TF, Olympus, Tokyo, Japan) [31,32,33,34] for approximately 2 to 7 days, depending on the growth rate of each individual strain, until they reached a concentration of 1–2 × 10^7^ cells/mL before immediate use for testing. The cells were enumerated via dark-field microscopy using a Neubauer counting chamber (Brand GmbH & Co. KG, Wertheim, Germany) according to the manufacturer’s recommendations, where a volume of 10 μL of *Borrelia* culture was counted in at least five squares at a 40× magnification. All tested strains are listed in Table 1.

### 2.2. Collection of Sera

Serum samples from eight volunteer blood donors were pooled for normal human serum (NHS), filtered through 0.22 µm syringe filters (Lab Logistics Group GmbH, Meckenheim, Germany), immediately aliquoted and frozen at −80 °C, and thawed only once before use. Heat-inactivated serum (HIS) was generated by incubating pooled NHS at 56 °C for 45 min, and this was used as a complement control [31,35]. Serum samples were tested for the presence of anti-*B. burgdorferi* s.l. IgM and IgG antibodies, and all were negative according to the ELISA assay (Euroimmun, Lübeck, Germany). To confirm the results, the sera were tested with the anti-*Borrelia* EUROLINE Western blot test (Euroimmun, Lübeck, Germany), and all samples were negative. Both commercial tests were used according to the manufacturer’s recommendations. While pooling was performed to minimise potential inter-individual fluctuations in complement activity and establish a standardised baseline for the assays, it should be noted that a pool derived from eight donors has a limited capacity to capture the full spectrum of genetic and physiological variations in human complement kinetics, which should be considered a limitation of the current investigation.

### 2.3. Ethical Statements

All healthy subjects gave their written informed consent at the Institute of Blood Transfusion and Haemobiology, Military Medical Academy, Belgrade, Republic of Serbia (decision number 1130-8, 20 November 2015). The study complied with the Declaration of Helsinki and was approved by the Ethics Committee of the Military Medical Academy, Belgrade, Republic of Serbia.

### 2.4. Serum Susceptibility

The serum susceptibility test in this study was performed as previously described by Wagemakers and colleagues [31]. Briefly, the spirochetes were counted under a dark-field microscope after incubation periods of one and three hours. In a 96-well microtiter plate (Thermo Scientific, Waltham, MA, USA), 25 μL of each *Borrelia* culture and 25 μL of NHS or HIS were mixed, and the plate was sealed and incubated at 33 °C for one and three hours. After incubation, 5 μL of the suspension from each well was examined under a dark-field microscope (BX41TF, Olympus, Tokyo, Japan). Samples were blinded, and 100 spirochetes per well were counted as either motile or immotile. To obtain consistent results and minimise the possibility of errors, the counting of motile and immotile spirochetes under the dark-field microscope was always performed by the same experienced person.

The loss of motility of spirochetes in NHS wells compared with HIS was indicative of complement-mediated killing and inactivation of spirochetes (susceptibility of *Borrelia* strains to NHS), while the presence of viable, motile spirochetes in NHS compared with HIS was indicative of the resistance of spirochetes to human complement [31]. However, it should be noted that a reduction or loss of spirochete motility in this assay may not strictly equate to immediate morphological cell lysis or definitive death. For each strain of *Borrelia*, the experiment was repeated at least three times; then, the median percentage of the number of viable and motile spirochetes was determined.

### 2.5. Statistical Analysis

A Kruskal–Wallis test was performed to identify the difference in motility among *Borrelia* species and among *Borrelia* strains within species observed in the serum susceptibility tests performed. The significance of the difference in motility between two strains, between two species in NHS, and between the two test conditions (NHS and HIS) for each *Borrelia* strain after one and three hours of incubation was analysed using the Mann–Whitney test. All statistical tests were considered statistically significant at the 0.05 probability level. Statistical analyses were performed using the program PASW Statistics 18 (IBM, Armonk, New York, USA). The graphs were created with Excel (Microsoft 365, Microsoft Corporation, Redmond, WA, USA).

## 3. Results

The range and median percentages of viable, motile spirochetes in the presence of NHS and HIS for each *Borrelia* strain after one hour (1 h) and three hours (3 h) of incubation are presented in Table 1. The most motile *Borrelia* species in the presence of NHS after three hours of incubation were *B. bavariensis* (median percentage of viable motile spirochetes = 100%, range = 96–100%), *B. afzelii* (median = 100%, range = 88–100%), and *B. valaisiana* (median = 76.5%; range = 52–89%), followed by *B. lusitaniae* (median = 1%, range = 0–50%) and *B. garinii* (median = 0%, range = 0–2%) (Table 1). All detailed raw data are presented in Supplementary Material Data S1.

**Table 1:** Range and median percentages of motile spirochetes for each *Borrelia burgdorferi* sensu lato strain originating from ticks (*Ixodes ricinus*) or humans in the presence of normal human serum (NHS) and heat-inactivated serum (HIS) after one (1 h) and three hours (3 h) of incubation showing resistance (R) or susceptibility (S).

Strain	Origin (Tick or Human)	P ^4^	NHS (1 h) Median (Range) ^1^ %	HIS (1 h) Median (Range) %	*p*-Value	NHS (3 h) Median (Range) %	HIS (3 h) Median (Range) %	*p*-Value	Susceptibility to NHS
*Borrelia valaisiana*									
RS 164_12b	Serbia (tick)	2	89 (85–91)	98 (98–98)	>0.05	87 (77–89)	97 (96–97)	>0.05	R ^2^
RS 224_10b	Serbia (tick)	3	75 (53–81)	97 (96–99)	**<0.05**	60 (52–76)	97 (95–97)	**<0.05**	S ^3^
median (range)			83 (53–91)	98 (96–99)		76.5 (52–89)	97 (95–97)		
*Borrelia afzelii*									
RS 71_11a	Serbia (tick)	3	100 (100–100)	100 (100–100)	>0.05	100 (100–100)	100 (100–100)	>0.05	R
RS 32_12b	Serbia (tick)	2	100 (100–100)	100 (100–100)	>0.05	100 (100–100)	100 (100–100)	>0.05	R
RS 230_13c	Serbia (tick)	3	100 (100–100)	100 (100–100)	>0.05	100 (100–100)	100 (100–100)	>0.05	R
RS 164_11a	Serbia (tick)	3	95 (94–98)	98 (98–98)	>0.05	93 (90–93)	97 (97–97)	>0.05	R
RS 168_11g	Serbia (tick)	2	97 (97–98)	99 (98–99)	>0.05	90 (88–94)	97 (94–97)	>0.05	R
RS 166_12a	Serbia (tick)	4	100 (100–100)	100 (100–100)	>0.05	100 (100–100)	100 (100–100)	>0.05	R
RS 168_11c	Serbia (tick)	2	100 (100–100)	100 (100–100)	>0.05	100 (100–100)	100 (100–100)	>0.05	R
RS 163_11i	Serbia (tick)	2	99 (98–100)	100 (99–100)	>0.05	99 (97–100)	99 (98–100)	>0.05	R
RS 167_11f	Serbia (tick)	4	100 (99–100)	100 (100–100)	>0.05	99 (98–100)	100 (99–100)	>0.05	R
RS 232_13b	Serbia (tick)	5	100 (99–100)	100 (100–100)	>0.05	99 (98–100)	100 (99–100)	>0.05	R
RS 168_11a	Serbia (tick)	4	100 (98–100)	99 (99–100)	>0.05	99 (98–100)	100 (99–100)	>0.05	R
RS 235_13cd	Serbia (tick)	5	100 (100–100)	100 (100–100)	>0.05	100 (100–100)	100 (100–100)	>0.05	R
median (range)			100 (94–100)	100 (98–100)		100 (88–100)	100 (94–100)		
*Borrelia garinii*									
RS 164_11g	Serbia (tick)	3	2 (0–3)	98 (97–99)	**<0.05**	2 (0–2)	98 (97–99)	**<0.05**	S
RS 226_10a	Serbia (tick)	4	0 (0–0)	100 (100–100)	**<0.05**	0 (0–0)	100 (100–100)	**<0.05**	S
median (range)			0 (0–3)	99.5 (97–100)		0 (0–2)	99.5 (97–100)		
*Borrelia bavariensis*									
RS 220_10e	Serbia (tick)	2	100 (100–100)	100 (100–100)	>0.05	100 (100–100)	100 (100–100)	>0.05	R
RS 160_13e	Serbia (tick)	3	100 (100–100)	100 (100–100)	>0.05	99 (96–100)	99 (99–99)	>0.05	R
RS 163_11h	Serbia (tick)	5	100 (100–100)	100 (100–100)	>0.05	99 (99–100)	100 (100–100)	>0.05	R
median (range)			100 (100–100)	100 (100–100)		100 (96–100)	100 (99–100)		
*Borrelia lusitaniae*									
RS 77_12b	Serbia (tick)	2	1 (0–2)	100 (100–100)	**<0.05**	1 (0–1)	99 (99–100)	**<0.05**	S
RS 226_10d	Serbia (tick)	2	20 (4–50)	100 (92–100)	**<0.05**	20 (3–45)	93 (91–94)	**<0.05**	S
RS 167_11c	Serbia (tick)	2	19 (15–20)	100 (100–100)	**<0.05**	1 (0–1)	100 (100–100)	**<0.05**	S
RS 162_11b	Serbia (tick)	4	12 (3–14)	100 (100–100)	**<0.05**	1 (1–2)	100 (100–100)	**<0.05**	S
RS 221_10c	Serbia (tick)	3	0 (0–7)	100 (100–100)	**<0.05**	0 (0–0)	100 (100–100)	**<0.05**	S
RS 167_11b	Serbia (tick)	2	0 (0–0)	100 (100–100)	**<0.05**	0 (0–0)	100 (98–100)	**<0.05**	S
RS 76_12a	Serbia (tick)	2	29 (26–35)	100 (100–100)	**<0.05**	15 (5–20)	100 (100–100)	**<0.05**	S
RS 222_10d	Serbia (tick)	2	0 (0–0)	100 (100–100)	**<0.05**	0 (0–0)	100 (100–100)	**<0.05**	S
PoHL1	Portugal (human)	6	53 (47–70)	100 (100–100)	**<0.05**	32 (5–50)	100 (80–100)	**<0.05**	S
Listu	Spain (tick)	7	10 (5–18)	100 (99–100)	**<0.05**	0 (0–3)	99 (98–100)	**<0.05**	S
Heavy	Spain (tick)	6	2 (0–5)	100 (100–100)	**<0.05**	0 (0–1)	100 (100–100)	**<0.05**	S
PotiB2	Portugal (tick)	7	1 (1–3)	100 (100–100)	**<0.05**	0 (0–1)	100 (100–100)	**<0.05**	S
median (range)			4.5 (0–70)	100 (92–100)		1 (0–50)	100 (80–100)		

*p*-values under 0.05 are in bold. ^1^ The median percentage of viable, motile spirochetes and the range were determined for each *Borrelia* strain in normal human serum (NHS) and heat-inactivated serum (HIS) after one and three hours of incubation. For each strain, the experiment was repeated at least three times. ^2^ R—*Borrelia* strains resistant to NHS after one and three hours of incubation; the presence of viable, motile spirochetes in NHS compared with HIS indicates the resistance of *Borrelia* strains to NHS. ^3^ S—*Borrelia* strains susceptible to NHS after one and three hours of incubation; the loss of motility of spirochetes in NHS compared with HIS indicates the susceptibility of *Borrelia* strains to NHS. ^4^ P—Total in vitro passage number.

**Figure 1:**
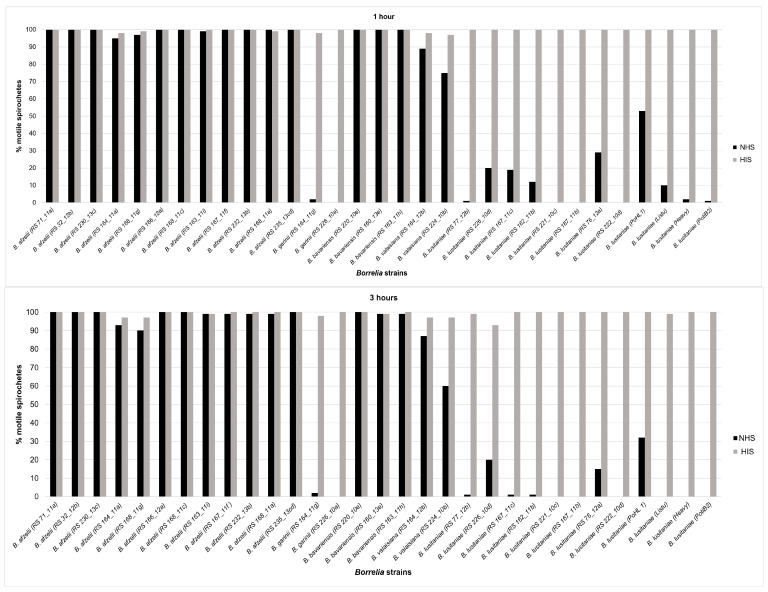
Serum susceptibility of 31 different *Borrelia burgdorferi* sensu lato strains and comparison of medians of motile and viable spirochetes in normal human serum (NHS) versus heat-inactivated serum (HIS) after one hour and three hours of incubation.

Based on the comparison of the median percentages of viable motile spirochetes in the presence of NHS and HIS, all tested *B. afzelii* strains (12/12), *B. bavariensis* strains (3/3), and 1/2 *B. valaisiana* strains (RS 164_12b) were resistant to NHS after one and three hours of incubation, while all tested *B. garinii* (2/2), all *B. lusitaniae* strains (12/12), and the other *B. valaisiana* strain (RS 224_10b) were susceptible to NHS after one and three hours of incubation (Table 1, Figure 1).

Intraspecies differences in motility were observed among the strains of *B. afzelii*, *B. valaisiana*, and *B. lusitaniae* after incubation with NHS (Figure 1). All tested *B. afzelii* strains exhibited a serum-resistant status, showing no significant loss of motility when incubated in NHS compared with HIS (*p* > 0.05). However, minor but statistically significant intraspecies variations in the baseline level of motility in NHS were noted, as two strains (RS 164_11a and RS 168_11g) differed significantly from the other *B.* *afzelii* isolates (*p* < 0.05). Differences in motility among the *B. valaisiana* strains were also observed after both one and three hours of incubation (*p* < 0.05). For *B. lusitaniae*, after one hour of incubation, there was a significant difference in motility between six strains (RS 226_10d, RS 167_11c, RS 162_11b, RS 76_12a, PoHL1, and Listu) and the other isolates (*p* < 0.05). After three hours of incubation, a significant difference in motility was observed between three *B. lusitaniae* strains (RS 226_10d, RS 76_12a, and PoHL1) and the other *B. lusitaniae* strains (*p* < 0.05). There were no significant differences in motility between the tick isolate from Serbia RS 226_10d and the human isolate from Portugal PoHL1 (*p* > 0.05), nor between the tick isolate from Serbia RS 76_12a and the human isolate from Portugal PoHL1 (*p* > 0.05) after three hours of incubation.

## 4. Discussion

The diversity of *B. burgdorferi* s.l. strains circulating in tick–mammal cycles in certain regions exceeds the diversity of strains that can cause LB in humans [36,37]. The ability of specific *Borrelia* strains to resist clearance by the host complement system is an important step for persistence in any host, including humans [10].

The assessment of the potential risk of tick-borne diseases (TBDs) in certain regions requires knowledge of tick presence and tick-borne pathogen (TBP) infection rates [36,37]. The region of Serbia is characterised by the high prevalence and diversity of *Borrelia* strains in *I. ricinus* and the dominance of *B. lusitaniae*, followed by *B. afzelii*, *B. bavariensis*, *B. garinii*, *B. valaisiana*, and *B. burgdorferi* s.s [16,17]. To elucidate the potential of local strains to establish a systemic infection in humans, we analysed the susceptibility of strains isolated from ticks to human complement in vitro using short-term incubation periods, which are epidemiologically essential to simulate the initial phase of transmission and assess the strains’ early viability.

There are several reliable methods for the differentiation between complement-affected and complement-unaffected cells [31,35,38]. For our study, we applied the counting of *Borrelia* using dark-field microscopy, as this is a reliable method for determining motile spirochetes [39].

However, some factors can influence *Borrelia* growth in vitro, such as medium ingredients, the pH of the medium, medium storage temperature, the temperature of incubation, contaminants, the sample’s cell density, the capacity of *Borrelia* species to grow, the number of different *Borrelia* cells in the sample, antecedent antibiotic therapy, the size of the skin biopsy specimens, the conditions of sample transport to the laboratory, etc. [32,33,34,40,41,42,43]. Although in vitro experiments have limiting factors, the method we used to assess the susceptibility of *Borrelia* to human serum based on motility in vitro is suitable for research, and this or a similar method was previously used by other authors [31,35,44,45].

We included well-established LB species (*B. afzelii*, *B. garinii*, and *B. bavariensis*) and the potentially pathogenic *B. lusitaniae*, alongside *B. valaisiana*, a species whose pathogenic status remains highly contested, all isolated from ticks collected at different regions in Serbia. We note as a limitation that the number of evaluated strains for *B. garinii*, *B. valaisiana*, and *B. bavariensis* is relatively limited. This sample size directly reflects the local field dynamics, where *B. lusitaniae* and *B. afzelii* are heavily dominant in *I. ricinus* ticks in Serbia, consequently yielding a higher number of available isolates. While our findings offer valuable insights into the local species-specific susceptibility trends, these patterns should be interpreted with caution given the smaller panel for the less prevalent species. The results showed that all *B. afzelii* strains were resistant to human complement in vitro. Although erythema migrans can be caused by several *B. burgdorferi* s.l. species (including the *B. garinii* and *B. bavariensis* species tested in this study), according to *Borrelia* skin culture results, erythema migrans in Europe is predominantly caused by *B. afzelii*, as well as acrodermatitis chronica atrophicans [1,2,7,8]. All *B. bavariensis* strains were also resistant to human complement, while two tested *B. garinii* strains were serum-susceptible. Both species are causative agents of LB and are mostly associated with neurological manifestations of LB [1,2,3]. The serum susceptibility/resistance pattern of LB species in vitro almost matches pathogenicity in humans, but *B. garinii* is an exception [11]. This species appears to be serum-susceptible in vitro, yet it is a confirmed, highly pathogenic causative agent of LB [1,2,11]. Similarly, *B. japonica* represents another contradiction to this pattern; although it exhibits distinct in vitro complement resistance, it is non-pathogenic to humans [11]. Together, these exceptions underscore the fundamental limitation of using in vitro serum resistance as a definitive proxy for human pathogenicity. As recently reviewed by Karami and colleagues [46], complement evasion is merely one of several parallel immune evasion mechanisms employed by *B. burgdorferi* s.l. strains. Other critical pathways, such as surface antigen variation (e.g., VlsE upregulation) and T-cell suppression, operate concurrently. Consequently, a strain like *B. garinii* may be eliminated by complement in vitro but can successfully bypass the host immune system and establish a systemic infection in vivo by exploiting these alternative evasion strategies.

The pathogenic potential of *B. valaisiana* has been highly debated and strongly questioned in recent years. There is historical evidence that *B. valaisiana* (previously referred to as genomic groups VS116 and M19) may also cause LB, although only skin biopsies or cerebrospinal fluid (CSF) samples from patients have been found to be positive for *B. valaisiana* DNA [47,48]. However, as comprehensively reviewed by Margos and colleagues [49], current evidence does not support human pathogenicity for this species, emphasising the absolute absence of any viable human isolates across Europe despite its widespread prevalence in questing ticks. In vitro, *B. valaisiana* appears to be susceptible to human complement [14], although Schwab and colleagues [13] showed that *B. valaisiana* strains differ in their susceptibility to human serum. It appears that serum-resistant *B. valaisiana* strains have different molecular mechanisms for inhibiting complement activation, which are independent of the recruitment of complement regulators or consist of the inactivation of central complement components. The exact molecular mechanism is still unclear. In our study, one of two *B. valaisiana* strains examined (strain RS 164_12b) (Table 1, Figure 1) was serum-resistant. This *B. valaisiana* strain is the second to show resistance to human complement, as one strain was first found in the study by Schwab and colleagues [13]. Further studies are needed to identify the mechanism behind the complement resistance of *B. valaisiana* strains circulating in this region.

*Borrelia lusitaniae* is considered to be a potentially pathogenic species [1,2]. The isolation of *B. lusitaniae* from chronic human skin lesions from Portugal [26] adds to the evidence that this species may cause LB. In the present study, we demonstrated that all *B. lusitaniae* strains from Serbia, Portugal, and Spain were serum-susceptible. This overall susceptibility agrees with Dieterich and colleagues [12], who evaluated a panel of *B. lusitaniae* isolates, including the human skin isolate PoHL1, and demonstrated their susceptibility to human complement. However, while the authors focused on long-term growth inhibition over a 10-day period, the objective of the current study was to determine the short-term viability profiles of circulating vector strains within their specific regional epidemiological context. To achieve this, survival profiles were evaluated after incubation periods of one and three hours, following the direct microscopic counting method described by Wagemakers and colleagues [31]. Utilising short-term incubation windows is advantageous because it simulates the initial phase when spirochetes encounter human complement immediately following transmission into the host bloodstream while preventing active bacterial replication from confounding the evaluation of early complement-mediated killing activity [14,31]. However, there was a statistically significant difference in motility among *B. lusitaniae* strains from Serbia, Portugal, and Spain in the presence of NHS after one and three hours of incubation (Table 1, Figure 1). Indeed, the data reveal a striking biological heterogeneity that challenges a simple binary classification. Instead of a uniform susceptibility profile, a distinct variation in tolerance thresholds was observed among the twelve tested strains. Furthermore, there were no statistically significant differences in motility between the two tick isolates from Serbia (RS 226_10d and RS 76_12a) and the human isolate from Portugal PoHL1 after three hours of incubation in the presence of NHS. The median percentages of viable, motile spirochetes of the two *B. lusitaniae* strains from Serbia (RS 226_10d and RS 76_12a) and the PoHL1 strain isolated from human skin were higher than those of other *B. lusitaniae* strains (Table 1). After three hours of incubation, the ranges and median percentages of motile spirochetes of these three strains were as follows: range = 3–45%, median = 20% (strain RS 226_10d); range = 5–20%, median = 15% (strain RS 76_12a); and range = 5–50%, median = 32% (strain PoHL1). In stark contrast, the remaining nine strains exhibited a near-complete loss of viability, with median motility values dropping to 0–1%. This clear divergence demonstrates that while all strains eventually decline, strains RS 226_10d, RS 76_12a, and PoHL1 display an intermediate capacity to withstand human complement activation compared to the rest of the group. This marked biological heterogeneity suggests that serum susceptibility within *B. lusitaniae* is a non-uniform characteristic, with certain strains demonstrating distinct survival dynamics. Interestingly, even within the serum-susceptible category, these two local Serbian isolates maintained a distinct level of motility that closely aligns with the higher survival observed in the human-derived isolate PoHL1. This heterogeneous behaviour among different strains is well-documented; as noted by Bhide and colleagues [35], complement susceptibility is not uniform, allowing for specific isolates within a susceptible group to exhibit enhanced short-term survival capacities. The similarity in activity between the local *Borrelia* strains and the human isolate from Portugal in the presence of NHS may suggest that some *B. lusitaniae* strains circulating in the region, if transmitted to humans, have the potential to stay viable and motile in the presence of human complement. The motility of local *B. lusitaniae* strains in the presence of human complement observed in this study should not be neglected since we recently detected and isolated a *B. lusitaniae* strain from the human blood of a patient with a clinical manifestation of LB—multiple erythema migrans [50]. Therefore, measuring survival after one and three hours in vitro is highly relevant, as it reflects this initial, critical phase in the bloodstream where certain local tick strains can maintain a level of motility matching that of a human pathogenic strain before final elimination. This critical clinical finding, combined with our in vitro data, strongly indicates that some strains of *B. lusitaniae* possessing these unique survival characteristics have the capacity for dissemination via the haematogenous route. Furthermore, for future studies, it is of particular interest to investigate the specific molecular mechanisms that allow these local *B. lusitaniae* strains to bypass early complement clearance and establish a systemic infection in humans.

A range of different factors affect infection with spirochetes in humans, with resistance to complement being only one of them. In Serbia, there are insufficient data on the *Borrelia* species that cause clinical manifestations of LB in humans. Our results suggest that some *Borrelia* strains that are resistant to human complement in vitro circulate in ticks in Serbia. Since overcoming the host complement system is an important step for persistence, it can be hypothesised that if these strains were transmitted, they could overcome the complement in the bloodstream in this region. However, as this in vitro resistance represents only one aspect of a multi-layered host immune response, these profiles must be interpreted with caution and do not directly imply the definitive capacity of these local isolates to establish a systemic infection in vivo.

## 5. Conclusions

In conclusion, *Borrelia* strains from ticks in Serbia exhibited differential in vitro susceptibility to human complement. All *B. afzelii* and *B. bavariensis* strains, and one *B. valaisiana* strain, were serum-resistant. However, the confirmed human pathogenicity of the former two species contrasts with the contested pathogenic status of *B. valaisiana*, which lacks human isolates.

Although diverse *Borrelia* strains circulate in this region, not all cause human disease. Some serum-resistant tick strains exhibit distinct survival profiles, indicating a potential to establish systemic infection, warranting regional clinical investigation. Nevertheless, this in vitro predictive potential is limited by parallel immune evasion mechanisms, as demonstrated by the serum-susceptible human pathogen *B. garinii*. Thus, in vitro susceptibility profiles alone cannot serve as a definitive proxy for clinical pathogenicity.

Statistical analysis showed that all *B. lusitaniae* strains from Serbia, Spain, and Portugal were serum-susceptible. However, complement significantly affected their motility differently, especially between the two Serbian tick isolates and the Portuguese human isolate. Prospective studies are required to elucidate the resistance mechanisms of the resistant *B. valaisiana* strain and to investigate how *B. lusitaniae* motility influences hematogenous dissemination. Furthermore, comparing the genomic characteristics of *B. lusitaniae* strains with different motility levels, particularly regarding outer membrane proteins, represents a promising avenue for future research. Additionally, future studies focused on confirming species-specific susceptibility patterns will require larger sample sizes for less prevalent species like *B. garinii*, *B. valaisiana*, and *B. bavariensis*.

## Data Availability

The original contributions presented in this study are included in the article/Supplementary Material. Further inquiries can be directed to the corresponding author.

## Supplementary Materials

The following supporting information can be downloaded at https://www.mdpi.com/article/10.3390/pathogens15070723/s1. Data S1: Raw data representing the counts of motile Borrelia cells from individual wells of microtiter plates after 1 and 3 h of incubation with NHS or HIS.
