# Supplementary material for: Resistance of Borrelia burgdorferi Sensu Lato Isolates from Serbia to Human Complement
Source: Pathogens. 2026 Jul 9;15(7):723. doi: 10.3390/pathogens15070723 (PMC13416043; doi:10.3390/pathogens15070723)
Supplement: Supplementary file 1 [file pathogens-15-00723-s001.zip › pathogens-4350211-supplementary.pdf]

Supplementary Material Data S1

The data below include *Borrelia* strains belonging to the species *Borrelia valaisiana*, *Borrelia afzelii*, *Borrelia lusitaniae*, *Borrelia bavariensis*, and *Borrelia garinii*, indicated by their respective codes.

Each number represents the count of motile *Borrelia* cells from a single well of a microtiter plate, recorded after 1 or 3 hours of culture incubation with NHS or HIS. The assays were performed either three times in triplicate (yielding a sequence of 9 numbers) or four times in triplicate (yielding a sequence of 12 numbers).

Abbreviations:

NHS (1h) – Normal human serum after 1 hour of incubation

NHS (3h) – Normal human serum after 3 hours of incubation

HIS (1h) – Heat-inactivated serum after 1 hour of incubation

HIS (3h) – Heat-inactivated serum after 3 hours of incubation

1. *Borrelia valaisiana*

- **RS 164_12b**
  - NHS (1h) → 85, 86, 87, 88, 88, 89, 89, 90, 90, 91, 91, 91
  - HIS (1h) → 98, 98, 98, 98, 98, 98, 98, 98, 98, 98, 98, 98
  - NHS (3h) → 77, 79, 81, 83, 85, 87, 87, 88, 88, 89, 89, 89
  - HIS (3h) → 96, 96, 96, 96, 96, 97, 97, 97, 97, 97, 97, 97
- **RS 224_10b**
  - NHS (1h) → 53, 57, 61, 66, 71, 75, 75, 77, 79, 80, 81, 81
  - HIS (1h) → 96, 96, 96, 96, 97, 97, 97, 98, 98, 99, 99, 99
  - NHS (3h) → 52, 54, 56, 58, 59, 60, 60, 63, 67, 71, 74, 76
  - HIS (3h) → 95, 95, 95, 96, 96, 97, 97, 97, 97, 97, 97, 97

2. *Borrelia afzelii*

- **RS 164_11a**
  - NHS (1h) → 94, 94, 94, 94, 94, 95, 95, 96, 96, 97, 98, 98

- HIS (1h) → 98, 98, 98, 98, 98, 98, 98, 98, 98, 98, 98, 98
- NHS (3h) → 90, 91, 91, 92, 92, 93, 93, 93, 93, 93, 93, 93
- HIS (3h) → 97, 97, 97, 97, 97, 97, 97, 97, 97, 97, 97, 97

- **RS 168_11g**
  - NHS (1h) → 97, 97, 97, 97, 97, 97, 97, 98, 98, 98, 98, 98
  - HIS (1h) → 98, 98, 98, 98, 98, 99, 99, 99, 99, 99, 99, 99
  - NHS (3h) → 88, 88, 89, 89, 89, 90, 90, 91, 92, 93, 94, 94
  - HIS (3h) → 94, 95, 95, 96, 96, 97, 97, 97, 97, 97, 97, 97
- **RS 71_11a**
  - NHS (1h) →100, 100, 100, 100, 100, 100, 100, 100, 100
  - HIS (1h) →100, 100, 100, 100, 100, 100, 100, 100, 100
  - NHS (3h) →100, 100, 100, 100, 100, 100, 100, 100, 100
  - HIS (3h) → 100, 100, 100, 100, 100, 100, 100, 100, 100

- **RS 32_12b**
  - NHS (1h) →100, 100, 100, 100, 100, 100, 100, 100, 100
  - HIS (1h) →100, 100, 100, 100, 100, 100, 100, 100, 100
  - NHS (3h) →100, 100, 100, 100, 100, 100, 100, 100, 100
  - HIS (3h) → 100, 100, 100, 100, 100, 100, 100, 100, 100

- **RS 230_13c**
  - NHS (1h) →100, 100, 100, 100, 100, 100, 100, 100, 100
  - HIS (1h) →100, 100, 100, 100, 100, 100, 100, 100, 100
  - NHS (3h) →100, 100, 100, 100, 100, 100, 100, 100, 100

    - HIS (3h) → 100, 100, 100, 100, 100, 100, 100, 100, 100
- **RS 166_12a**
    - NHS (1h) →100, 100, 100, 100, 100, 100, 100, 100, 100
    - HIS (1h) →100, 100, 100, 100, 100, 100, 100, 100, 100
    - NHS (3h) →100, 100, 100, 100, 100, 100, 100, 100, 100
    - HIS (3h) → 100, 100, 100, 100, 100, 100, 100, 100, 100
- **RS 168_11c**
    - NHS (1h) →100, 100, 100, 100, 100, 100, 100, 100, 100
    - HIS (1h) →100, 100, 100, 100, 100, 100, 100, 100, 100
    - NHS (3h) →100, 100, 100, 100, 100, 100, 100, 100, 100
    - HIS (3h) → 100, 100, 100, 100, 100, 100, 100, 100, 100
- **RS 163_11i**
    - NHS (1h) → 98, 98, 99, 99, 99, 100, 100, 100, 100
    - HIS (1h) → 99, 99, 100, 100, 100, 100, 100, 100, 100
    - NHS (3h) → 97, 98, 98, 99, 99, 100, 100, 100, 100
    - HIS (3h) → 98, 98, 99, 99, 99, 100, 100, 100, 100
- **RS 167_11f**
    - NHS (1h) → 99, 99, 99, 100, 100, 100, 100, 100, 100
    - HIS (1h) → 100, 100, 100, 100, 100, 100, 100, 100, 100
    - NHS (3h) → 98, 98, 98, 99, 99, 100, 100, 100, 100
    - HIS (3h) → 99, 99, 100, 100, 100, 100, 100, 100, 100

- **RS 232_13b**
    - NHS (1h) →99, 99, 99, 99, 100, 100, 100, 100, 100
    - HIS (1h) →100, 100, 100, 100, 100, 100, 100, 100, 100
    - NHS (3h) → 98, 99, 99, 99, 99, 100, 100, 100, 100
    - HIS (3h) → 99, 100, 100, 100, 100, 100, 100, 100, 100
- **RS 168_11a**
    - NHS (1h) → 98, 99, 99, 100, 100, 100, 100, 100, 100
    - HIS (1h) → 99, 99, 99, 99, 99, 100, 100, 100, 100
    - NHS (3h) → 98, 99, 99, 99, 99, 100, 100, 100, 100
    - HIS (3h) → 99, 99, 100, 100, 100, 100, 100, 100, 100
- **RS 235_13cd**
    - NHS (1h) →100, 100, 100, 100, 100, 100, 100, 100, 100
    - HIS (1h) →100, 100, 100, 100, 100, 100, 100, 100, 100
    - NHS (3h) →100, 100, 100, 100, 100, 100, 100, 100, 100
    - HIS (3h) → 100, 100, 100, 100, 100, 100, 100, 100, 100

3. ***Borrelia garinii***

- **RS 164_11g**
    - NHS (1h) → 0, 0, 1, 1, 2, 2, 3, 3, 3
    - HIS (1h) → 97, 97, 98, 98, 98, 99, 99, 99, 99
    - NHS (3h) → 0, 0, 1, 1, 2, 2, 2, 2, 2
    - HIS (3h) → 97, 97, 98, 98, 98, 99, 99, 99, 99
- **RS 226_10a**
    - NHS (1h) → 0, 0, 0, 0, 0, 0, 0, 0, 0
    - HIS (1h) →100, 100, 100, 100, 100, 100, 100, 100, 100
    - NHS (3h) → 0, 0, 0, 0, 0, 0, 0, 0, 0
    - NIH (3h) → 100, 100, 100, 100, 100, 100, 100, 100, 100

4. *Borrelia bavariensis*

- **RS 220_10e**
  - NHS (1h) →100, 100, 100, 100, 100, 100, 100, 100, 100
  - HIS (1h) →100, 100, 100, 100, 100, 100, 100, 100, 100
  - NHS (3h) →100, 100, 100, 100, 100, 100, 100, 100, 100
  - HIS (3h) → 100, 100, 100, 100, 100, 100, 100, 100, 100

- **RS 160_13e**
  - NHS (1h) → 100, 100, 100, 100, 100, 100, 100, 100, 100
  - HIS (1h) → 100, 100, 100, 100, 100, 100, 100, 100, 100
  - NHS (3h) → 96, 97, 98, 98, 99, 99, 100, 100, 100
  - HIS (3h) → 99, 99, 99, 99, 99, 99, 99, 99, 99

- **RS 163_11h**
  - NHS (1h) → 100, 100, 100, 100, 100, 100, 100, 100, 100
  - HIS (1h) → 100, 100, 100, 100, 100, 100, 100, 100, 100
  - NHS (3h) → 99, 99, 99, 99, 99, 100, 100, 100, 100
  - HIS (3h) → 100, 100, 100, 100, 100, 100, 100, 100, 100

5. *Borrelia lusitaniae*

RS 77_12b

- NHS (1h) → 0, 0, 0, 1, 1, 1, 1, 1, 1, 2, 2, 2
- HIS (1h) → 100, 100, 100, 100, 100, 100, 100, 100, 100, 100, 100, 100
- NHS (3h) → 0, 0, 0, 0, 1, 1, 1, 1, 1, 1, 1, 1
- HIS (3h) → 99, 99, 99, 99, 99, 99, 100, 100, 100, 100, 100, 100

RS 226_10d

- NHS (1h) → 4, 7, 10, 13, 17, 20, 20, 25, 31, 38, 44, 50
- HIS (1h) → 92, 94, 95, 97, 98, 100, 100, 100, 100, 100, 100, 100
- NHS (3h) → 3, 3, 3, 3, 3, 20, 20, 45, 45, 45, 45, 45
- HIS (3h) → 91, 91, 92, 92, 92, 93, 93, 93, 94, 94, 94, 94

RS 167_11c

- NHS (1h) → 15, 16, 17, 18, 19, 19, 19, 19, 19, 20, 20, 20
- HIS (1h) → 100, 100, 100, 100, 100, 100, 100, 100, 100, 100, 100, 100
- NHS (3h) → 0, 0, 0, 0, 1, 1, 1, 1, 1, 1, 1, 1
- HIS (3h) → 100, 100, 100, 100, 100, 100, 100, 100, 100, 100, 100, 100

RS 162_11b

- NHS (1h) → 3, 4, 7, 9, 10, 12, 12, 13, 13, 14, 14, 14
- HIS (1h) → 100, 100, 100, 100, 100, 100, 100, 100, 100, 100, 100, 100
- NHS (3h) → 1, 1, 1, 1, 1, 1, 1, 2, 2, 2, 2, 2
- HIS (3h) → 100, 100, 100, 100, 100, 100, 100, 100, 100, 100, 100, 100

RS 221_10c

- NHS (1h) → 0, 0, 0, 0, 0, 0, 0, 0, 1, 3, 4, 7
- HIS (1h) → 100, 100, 100, 100, 100, 100, 100, 100, 100, 100, 100, 100
- NHS (3h) → 0, 0, 0, 0, 0, 0, 0, 0, 0, 0, 0, 0
- HIS (3h) → 100, 100, 100, 100, 100, 100, 100, 100, 100, 100, 100, 100

RS 167_11b

- NHS (1h) → 0, 0, 0, 0, 0, 0, 0, 0, 0, 0, 0, 0
- HIS (1h) → 100, 100, 100, 100, 100, 100, 100, 100, 100, 100, 100, 100
- NHS (3h) → 0, 0, 0, 0, 0, 0, 0, 0, 0, 0, 0, 0
- HIS (3h) → 98, 98, 99, 99, 100, 100, 100, 100, 100, 100, 100, 100

RS 76_12a

- NHS (1h) → 26, 26, 27, 27, 28, 29, 29, 31, 32, 34, 35, 35
- HIS (1h) → 100, 100, 100, 100, 100, 100, 100, 100, 100, 100, 100, 100
- NHS (3h) → 5, 5, 5, 5, 5, 15, 15, 20, 20, 20, 20, 20
- HIS (3h) → 100, 100, 100, 100, 100, 100, 100, 100, 100, 100, 100, 100

RS 222_10d

- NHS (1h) → 0, 0, 0, 0, 0, 0, 0, 0, 0, 0, 0, 0
- HIS (1h) → 100, 100, 100, 100, 100, 100, 100, 100, 100, 100, 100, 100
- NHS (3h) → 0, 0, 0, 0, 0, 0, 0, 0, 0, 0, 0, 0
- HIS (3h) → 100, 100, 100, 100, 100, 100, 100, 100, 100, 100, 100, 100

POHL1

- NHS (1h) → 47, 48, 49, 51, 52, 53, 53, 58, 62, 65, 68, 70
- HIS (1h) → 100, 100, 100, 100, 100, 100, 100, 100, 100, 100, 100, 100
- NHS (3h) → 5, 5, 5, 5, 5, 27, 37, 37, 37, 37, 37, 50
- NHS (3h) → 80, 85, 90, 92, 95, 100, 100, 100, 100, 100, 100, 100

Listu

- NHS (1h) → 5, 6, 7, 8, 9, 10, 10, 12, 14, 15, 17, 18
- HIS (1h) → 99, 99, 99, 99, 99, 100, 100, 100, 100, 100, 100, 100

- NHS (3h) → 0, 0, 0, 0, 0, 0, 0, 1, 1, 2, 2, 3
- HIS (3h) → 98, 98, 98, 98, 98, 99, 99, 99, 100, 100, 100, 100

Heavy

- NHS (1h) → 0, 0, 1, 1, 1, 2, 2, 3, 3, 4, 5, 5
- HIS (1h) → 100, 100, 100, 100, 100, 100, 100, 100, 100, 100, 100, 100
- NHS (3h) → 0, 0, 0, 0, 0, 0, 0, 0, 0, 1, 1, 1
- HIS (3h) → 100, 100, 100, 100, 100, 100, 100, 100, 100, 100, 100, 100

PotiB2

- NHS (1h) → 1, 1, 1, 1, 1, 1, 1, 2, 2, 2, 3, 3
- HIS (1h) → 100, 100, 100, 100, 100, 100, 100, 100, 100, 100, 100, 100
- NHS (3h) → 0, 0, 0, 0, 0, 0, 0, 0, 0, 1, 1, 1
- HIS (3h) → 100, 100, 100, 100, 100, 100, 100, 100, 100, 100, 100, 100
